# Faunal Deposits in Pre-Beaker Funerary Contexts at Humanejos (Parla, Madrid, Spain)

**DOI:** 10.3390/ani16172653

**Published:** 2026-08-24

**Authors:** Verónica Estaca-Gómez, Laura Aguilar-Molina, Ana Mercedes Herrero-Corral, Rodrigo Paulos-Bravo, Ruth Maicas, Raúl Flores-Fernández, Rafael Garrido-Pena, José Yravedra

**Affiliations:** 1 Department of Prehistory, Ancient History and Archaeology, Complutense University of Madrid, C/ Profesor Aranguren sn, 28040 Madrid, Spain; vestaca@ucm.es (V.E.-G.); laguilmo109@alumnes.ub.edu (L.A.-M.); 2 Research Group Arqueología Prehistórica, Department of Prehistory, Ancient History and Archaeology, Complutense University of Madrid, C/ Profesor Aranguren sn, 28040 Madrid, Spain; 3 Department of Prehistory and Archaeology, University of Barcelona, Gran Vía de las Corts Catalanes 585, 08007 Barcelona, Spain; 4 Ramón y Cajal Research Fellow, Department of Archaeology and Social Processes, Institute of History (IH), Centre for Human and Social Sciences (CCHS), Spanish National Research Council (CSIC), C. de Albasanz 26, 28037 Madrid, Spain; ana.herrero@cchs.csic.es; 5 Research Support Center (CAI) of Archaeometry, and Archaeological Analysis, Complutense University of Madrid, C/ Profesor Aranguren SN, 28040 Madrid, Spain; 6 Department of Prehistory, Museo Arqueológico Nacional, C/Serrano 13, 28001 Madrid, Spain; ruth.maicas@cultura.gob.es; 7 Territorial Archaeologist, Territorial Service of Culture, Tourism and Sport of León, Junta de Castilla y León, Av. Peregrios, sn, 24008 León, Spain; 8 Department of Prehistory and Archaeology, Autonomous University of Madrid, Calle de Francisco Tomás y Valiente, 9, 28049 Madrid, Spain; 9 Research Group Ecosistemas Cuaternarios C/ Profesor Aranguren sn, 28040 Madrid, Spain

**Keywords:** faunal deposits, pre-Beaker Copper Age, zooarchaeology

## Abstract

This study presents the first zooarchaeological analysis of the faunal remains associated with the pre-Beaker funerary contexts of Humanejos (Parla, Madrid, Spain). A total of 3974 animal remains recovered from 31 burial pits were analysed. Species representation was assessed using the NISP and MNI, and bone surface modifications were examined to evaluate human activities. The results indicate that domestic taxa account for 84% of the total MNI and that the faunal remains occur in two distinct types of assemblages. The first consists of complete or nearly complete articulated animals intentionally deposited within the graves, including bovine skulls and the interment of dogs, pigs, and caprines. The second comprises disarticulated faunal remains representing one or several individuals, with no apparent anatomical association, and is interpreted as refuse or pit-fill material. This assemblage exhibits greater taxonomic diversity, with caprines predominating, and provides evidence of carcass processing and consumption, as indicated by cut marks. The differences between these assemblages offer new insights into the role of animals in pre-Beaker funerary practices at Humanejos and contribute to a better understanding of the relationship between funerary rituals, animal management, and daily life in Copper Age central Iberia.

## Simple Summary

In this paper, we present the zooarchaeological and taphonomic study of faunal remains from 31 burial pits of the pre-Bell Beaker funerary contexts of Humanejos (Parla, Madrid, Spain). The assemblage was dominated by animals, and sheep/goats, cattle and pigs were the most common species. Two different types of faunal assemblages were identified. Some consisted of complete or nearly complete articulated animals intentionally placed in the graves, including cattle skulls, dogs, pigs and sheep/goats. Others were composed of disarticulated remains bearing traces of processing and consumption, suggesting that they represent refuse incorporated into the pit fills. These findings provide new insights into the role of animals in Copper Age funerary practices in central Iberia and contribute to a better understanding of the relationships between funerary rituals, animal exploitation, and everyday life.

## 1. Introduction

Until the end of the twentieth century, zooarchaeological studies of the Copper Age in the inland Iberian Peninsula were scarce. Only a limited number of archaeological sites had been investigated, and the available faunal assemblages were generally small. In addition, many of these sites presented stratigraphic problems, with faunal remains originating from different chrono-cultural contexts. As a result, the zooarchaeological evidence available for this period was extremely limited [1,2].

In recent decades, the growing number of archaeological interventions, many of them undertaken within the framework of preventive archaeology, has considerably expanded the zooarchaeological record of the Copper Age in the Iberian Peninsula. Numerous sites in the Madrid region, including Humanejos, Camino de las Yeseras, Barrio del Castillo, Aldovea, Soto de Henares, Zanjillas, La Cuesta, El Espinillo, El Juncal, Huerta de los Cabreros and Las Cabeceras [3,4,5,6,7,8,9,10,11,12,13,14,15,16], together with sites in Guadalajara, Ciudad Real, Valladolid, Burgos, southern Spain and Portugal [17,18,19,20,21,22,23,24,25,26,27,28,29,30,31,32], have provided a substantial body of new zooarchaeological and taphonomic evidence. As a result, our understanding of animal husbandry, exploitation strategies and the role of animals in Copper Age societies has improved significantly. Moreover, some of these studies have moved beyond the traditional identification and description of faunal assemblages by incorporating new analytical and interdisciplinary approaches into zooarchaeological research [33,34,35].

Based on these studies, Copper Age zooarchaeological contexts in the Iberian Peninsula represent a highly complex archaeological record [7,16,28,36,37,38,39,40]. Faunal remains appear mainly in two types of assemblages: those consistent with the secondary deposition of domestic refuse and those resulting from intentional deposits.

Domestic refuse is characterized by fragmented and anatomically disarticulated remains displaying bone surface modifications related to food processing and other activities such as skinning. These faunal remains are mainly composed of sheep/goats, cattle and pigs, while dogs and wild animals occur less frequently [5,12,13,14,15,16,18,21,25,27,28,34]. These species formed the basis of Copper Age animal husbandry. Such assemblages are generally interpreted from an economic perspective.

In intentional deposits, complete or partially complete animals were deliberately interred, usually in anatomical connection, and their bones are rarely fractured. These faunal remains tend to be dominated by dogs, pigs and cattle, although other taxa, such as caprines, may occasionally be present [13,14,15,16,28,33,37,38,40,41]. Intentional deposits also tend to include a higher proportion of juvenile individuals, under 4–5 years of age, whereas domestic bone assemblages generally display mortality profiles dominated by adults [7,13,14,15,16,28,33,40,41].

The interpretation of faunal remains becomes even more challenging when they are associated with human burials. Although relatively uncommon, such associations have been documented in Copper Age contexts across the Iberian Peninsula, both in caves [28] and at open-air sites [37,38,39,40,41,42,43,44]. Their archaeological interpretation remains controversial because the association of faunal remains with burials does not necessarily imply intentional deposition. While some assemblages may represent grave goods, offerings associated with funerary rituals, or other forms of symbolic behaviour, others may simply reflect the incorporation of animal bones in the fills of burial pits [28,36,45,46,47,48]. Distinguishing between these different depositional processes is therefore essential for understanding the role of animals in Copper Age funerary practices.

This paper presents the first zooarchaeological study of the faunal remains associated with the pre-Beaker funerary contexts of Humanejos. By combining taxonomic composition, mortality profiles, anatomical representation and taphonomic evidence, this study aims to distinguish intentional animal deposits from faunal remains incorporated into burial fills that are consistent with the secondary deposition of domestic refuse, thereby providing a more comprehensive interpretation of the role of animals in Copper Age funerary contexts.

## 2. The Site

**Figure 1:**
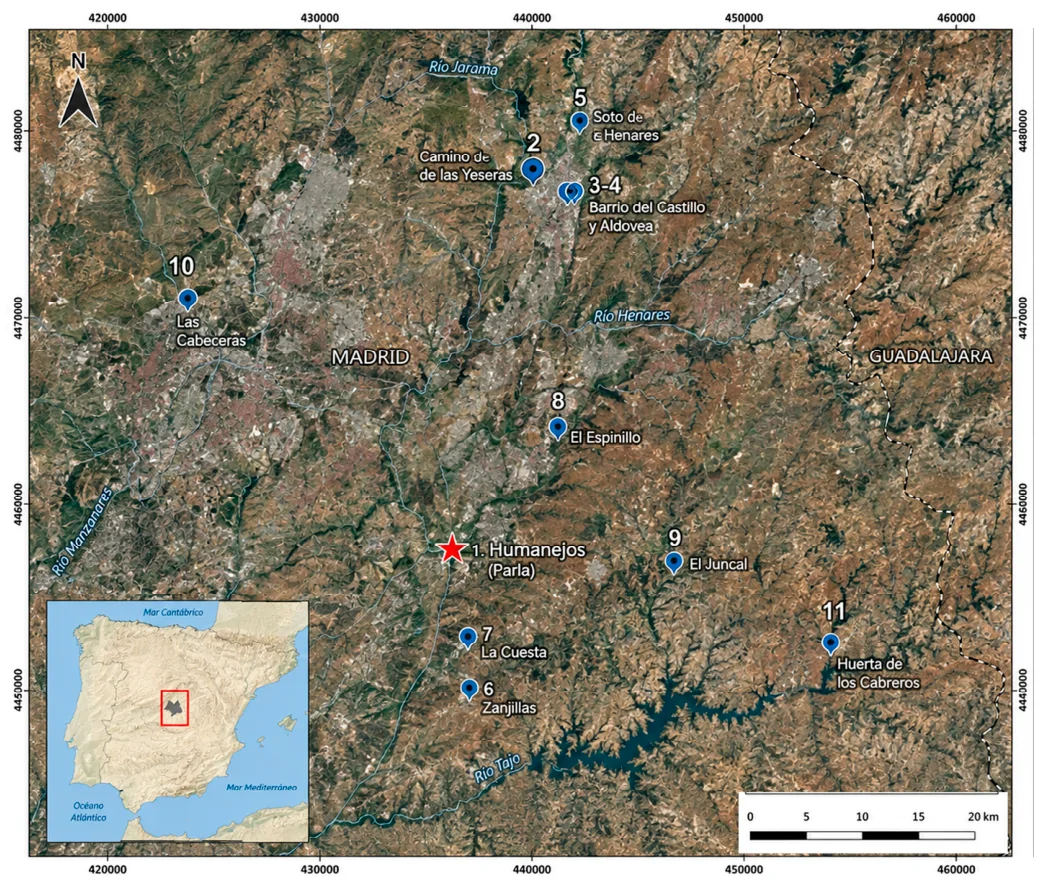
Location of Humanejos and other pre-Beaker Chalcolithic sites within the regional framework of the Community of Madrid mentioned in the text.

Humanejos is situated in Parla, south of Madrid (Spain), and takes its name from a nearby stream that flows into the Tagus River (Figure 1). The site was first identified in 1982 during an emergency archaeological intervention directed by Guillermo Kurtz in advance of the construction of the southern junction of the N-401 road. However, the archaeological significance and extent of the site were not fully recognized until 2002, when archaeological monitoring associated with a major urban development project prompted extensive preventive excavations. These investigations revealed a large archaeological complex currently covering approximately 20 ha, including both settlement areas and funerary contexts. The earliest occupations date to the Late Neolithic (4th millennium BCE), followed by phases corresponding to the Copper Age, Early Bronze Age (Protocogotas) and Iron Age, as well as later Roman and Medieval occupations [49].

The most prolific period at Humanejos is represented by the pre-Beaker phases, namely the Late Neolithic (3300–2900 cal BC) and Copper Age (c. 2900–2500 cal BC), together with Beaker contexts (c. 2500–2000 cal BC). The faunal assemblage examined in this study derives from pre-Beaker burials. A total of 44 inhumations have been documented for this period, of which 31 contained faunal remains. These exhibit considerable variability, comprising single, double and multiple human interments (24, 8 and 9 cases, respectively). Individuals of both sexes are represented, with adults predominating over subadults, and strontium isotope analyses indicate that some non-local individuals were also buried at the site [50,51]. Together with the faunal deposits discussed in this article, the grave goods associated with these burials include globular pottery vessels with inward-curving rims and lithic artefacts such as retouched flint blades, arrowheads and a dagger/knife showing varying degrees of use-wear [52]. From c. 2700–2600 cal BC onwards, copper objects, including awls, daggers/knives, axes and variscite beads, also occur.

## 3. Sample and Methods

As in most studies of Iberian Prehistory, the criteria used here to identify intentional animal deposits are based on approaches developed through debates concerning the archaeological recognition of symbolic behaviour beyond purely functional activities. These criteria derive from comparisons between ordinary domestic assemblages and unusual faunal occurrences. In this perspective, neither the faunal remains nor the archaeological context alone define a deposit as symbolic. Rather, their interpretation depends on the combined assessment of contextual, anatomical, and taphonomic evidence, since such practices were embedded within the social and cultural dynamics of prehistoric communities [53,54,55].

Accordingly, following Grant and Richards [56] and Thomas [57], intentional animal deposits are understood as deliberate burials of complete animals, isolated crania, or selected anatomical elements deposited in anatomical connection. These remains typically show evidence of intentional selection and occur recurrently within specific archaeological contexts. Conversely, fragmented and disarticulated faunal remains displaying characteristics typical of domestic refuse, such as extensive fragmentation, evidence of butchery or food consumption, and the absence of anatomical connection, were considered likely to derive from the backfilling of burial pits rather than from intentional funerary deposits. Therefore, the distinction between intentional animal deposits and faunal remains consistent with the secondary deposition of domestic refuse was based on the combined assessment of the archaeological context, anatomical representation, articulation, taphonomic modifications, and mortality profiles.

The sample analysed in this study comprises 3948 faunal remains. The assemblage associated with the funerary pits of Humanejos was classified into two broad categories. The first consists of faunal remains incorporated into the pit fills, ranging from a few isolated fragments to relatively large faunal assemblages, which are considered consistent with the secondary deposition of domestic refuse. The second comprises intentionally deposited remains, including complete animals, partially articulated body portions, bovine crania and deer antlers.

Faunal remains were identified through comparison with reference collections housed at the Universidad Complutense de Madrid and with the aid of the atlases published by Barone, Schmid, Martin and Blázquez, and Hilson [58,59,60]. To distinguish between specific taxa, such as *Ovis aries*, *Capra hircus*, *Capra pyrenaica* and *Capreolus capreolus*, the methods described by Boesseneck, Prummel and Frisch, and Fernández were employed [61,62,63,64,65]. The criteria proposed by Prummel [66] were followed to distinguish between *Cervus elaphus* and *Bos taurus*. The distinction between dogs and wolves was based on osteometric criteria, with shoulder height estimated from the maximum length of the long bones, following Koudelka [67].

Species abundance was estimated using the NISP (Number of Identified Specimens) and the MNI (Minimum Number of Individuals), following Brain [68]. Mortality patterns were reconstructed through dental eruption, tooth wear and epiphyseal fusion data. We used the criteria proposed by Silver [69], Payne [70,71], Grant [72], Pérez-Ripoll [73], Mariezkurrena [74], Brown and Chapman [75,76], Klein et al. [77], Levine [78], Guadelli [79], Rowlett and Chiu [80], Hambleton [81], Azorit Casas [82] and Marín et al. [83]. The resulting data were grouped into three age categories: infant, juvenile–subadult and adult–senile.

The bone surfaces of all specimens were examined using 10–15× magnification, following Blumenschine [84], to identify bone surface modifications and traces of human activity. Cut marks were identified and differentiated from other modifications, such as trampling marks and carnivore damage, following the criteria proposed by Binford [85], Fernández-Jalvo and Andrews [86], and Yravedra [87]. Weathering stages and root etching alterations were recorded according to Behrensmeyer [88] and Fernández-Jalvo and Andrews [86].

## 4. Results

Of the 44 pre-Beaker burial pits identified at Humanejos, 31 (68.9%) contained faunal remains. Among the burials with faunal evidence, 71% contained only animal remains incorporated into the grave fills, whereas 29% included intentional animal deposits placed alongside the deceased (Table 1).

The analysed assemblage comprises 3948 faunal remains. According to the NISP, *Ovis*/*Capra* are the most abundant taxon, followed by *Bos taurus*, *Sus* sp., *Canis familiaris*, and wild species (Table 2). In terms of the MNI, caprines, both *Ovis aries* and *Capra hircus*, predominate (37.8%), followed by *Bos taurus* (14.9%), *Sus* sp. (16.9%) and *Canis familiaris* (8.0%). Other identified taxa include *Cervus elaphus* (10.0%), *Oryctolagus cuniculus* (6.0%), *Equus ferus* (6.0%), and *Capreolus capreolus*, represented by a single individual.

The distribution of taxa across the funerary pits further highlights the importance of *Ovis*/*Capra*, which occur in 83.9% of the burials containing faunal remains. *Bos taurus* are present in 71% of these contexts, *Sus* sp. in 58.1%, and *Canis familiaris* in 48.4% (Table 2). These data suggest that animal husbandry was primarily focused on caprines, with cattle and pigs also playing a significant role. Wild taxa indicate that hunting continued to contribute to subsistence practices during the Copper Age. *Cervus elaphus* was identified in 51.6% of the burials, whereas *Equus ferus* occurred in 38.7% (Table 2).

A minimum of 201 individuals was identified in the assemblage. Age at death could be estimated for 183 of them. Adult individuals predominate, accounting for 75.6% of the total sample. The remaining individuals comprise infants (13.4%) and juveniles–subadults (10.9%). *Ovis*/*Capra* and *Sus* sp. display the greatest variability in mortality profiles, with 36.8% and 44.1%, respectively, falling within the non-adult categories.

Before discussing skeletal representation in detail, it is important to note that bone preservation is generally good. Most specimens exhibit low weathering stages, with Behrensmeyer stages 1–2 predominating [88], indicating relatively rapid burial. Biochemical alterations and vermiculations are more frequent, affecting most of the assemblage to a low or moderate degree. Nevertheless, these alterations have not hindered the observation of bone surfaces or the identification of bone surface modifications, such as carnivore and human alterations. Carnivore tooth marks are concentrated on the epiphyses of long bones and on axial elements, frequently causing the collapse of epiphyseal ends.

Cut marks were recorded on all taxa represented within the refuse assemblages, including both domestic and wild species, providing clear evidence of carcass-processing and consumption practices (Table 3). No cut-marked specimens were identified in *Canis familiaris*. In ungulates, cut marks on long bones are mainly associated with defleshing the humerus and femur, whereas those located around the joints indicate disarticulation. In some cases, their distribution also indicates the exploitation of specific anatomical parts, such as the removal of cheek meat from the mandible (Table 3).

**Figure 2:**
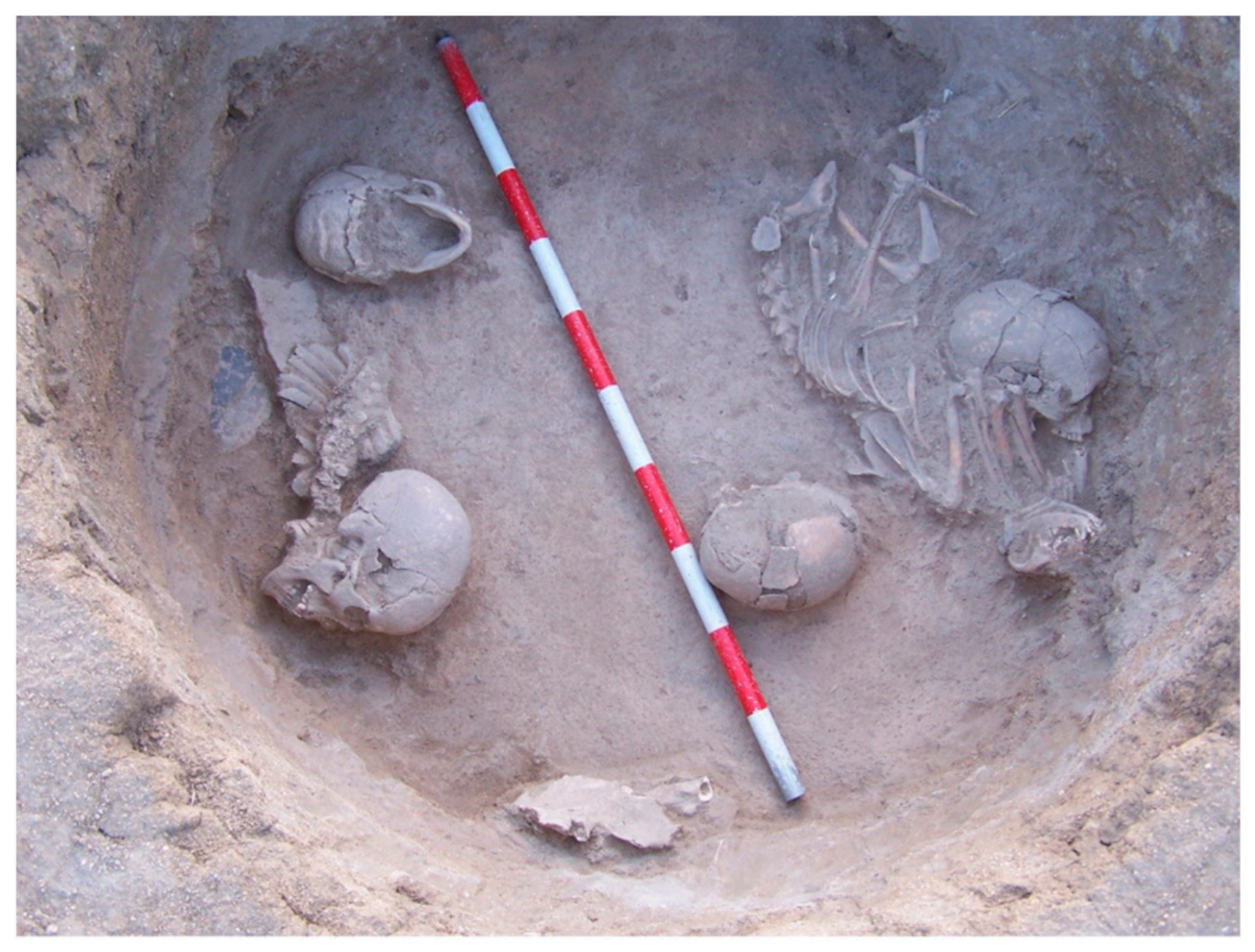
Dog burial between human skulls in pit 35. Photograph Sara Genicio.

**Figure 3:**
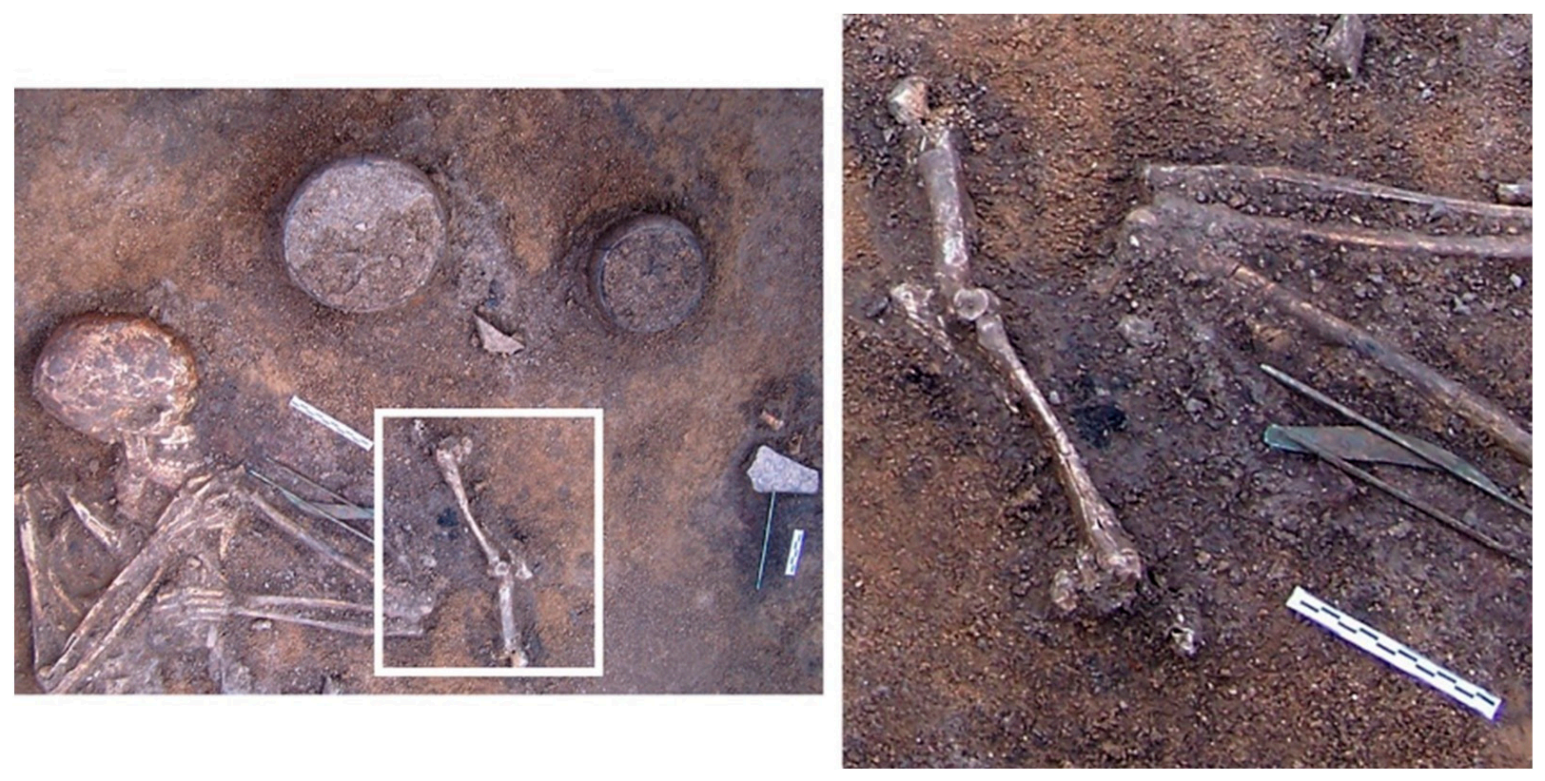
Burial pit 66, with a caprine forelimb among grave goods. Photograph Sara Genicio.

**Figure 4:**
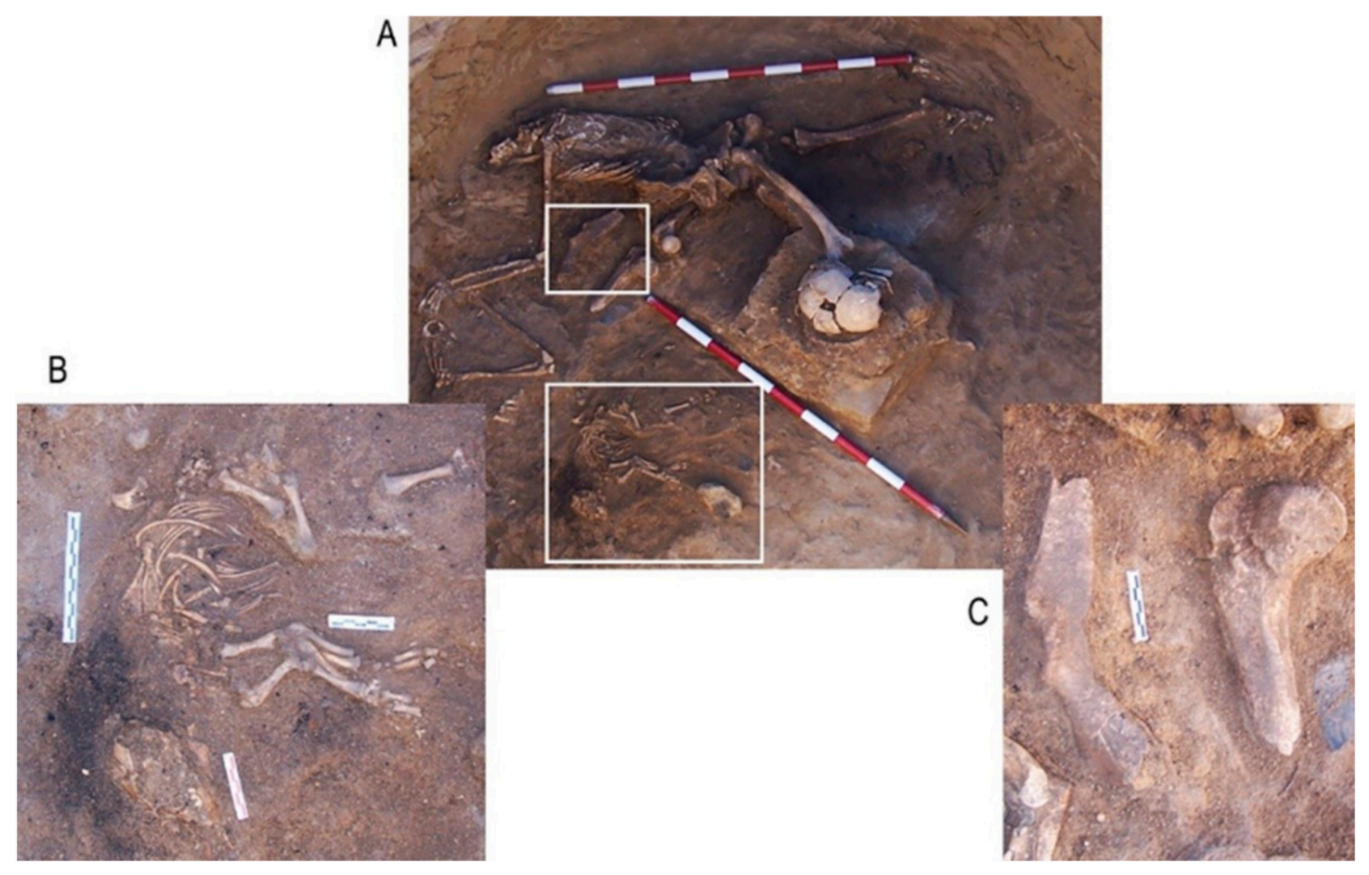
Burial pit 60, with an adult human male (**A**), a piglet (**B**), in upper white box and a bovine pelvic in the second white box. (**C**). Photograph Sara Genicio.

Intentional deposits (Table 4) include four cattle skulls (burials 25, 60, 62), three whole dogs (burials 35, 43, 53, Figure 2), one caprine (burial 20), one articulated caprine forelimb (humerus, radius, ulna) (burial 66, Figure 3) and a piglet (burial 60) (Figure 4). These remains are notable for the absence of cut marks and other anthropogenic modifications associated with food consumption, in contrast to the refuse assemblages. The significance of these intentionally deposited animal remains is difficult to interpret, as they are associated with the burials of human individuals of different age groups, who are not always represented by complete skeletal remains but instead by selected elements, most commonly the skull (Figure 2). Nor was a consistent pattern observed in relation to grave goods, as intentional animal deposits occur in burials both with and without such items.

Thus, cattle skulls occur in different archaeological contexts: associated with antlers (graves 25 and 62), with pottery (grave 62), or without associated pottery vessels (graves 25 and 60). Similarly, the caprine deposit may accompany the deceased together with a substantial assemblage of pottery vessel and copper artefacts (grave 66; Figure 3) or be associated with a dagger and two flint points. The dog burials also display considerable variability. One individual was associated with a single pottery vessel (grave 35), another with numerous pottery vessels (grave 53), whereas a third was accompanied only by a flint point and no pottery (grave 43). These differences indicate that intentional animal deposits did not follow a single funerary pattern.

**Figure 5:**
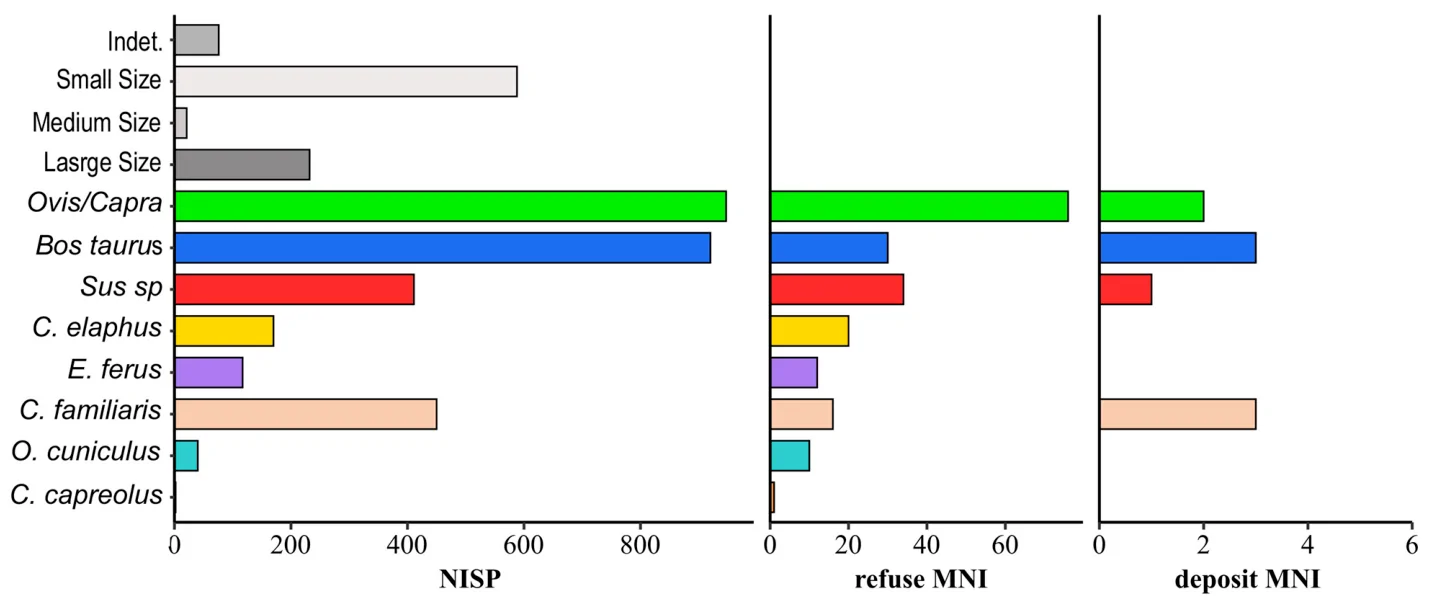
Faunal representation by the NISP and MNI in refuse fills and intentional deposits.

Nevertheless, the taxonomic composition and taphonomic evidence described above show that dogs and cattle predominate among the intentional deposits associated with human burials. This contrasts with the faunal assemblages recovered from the refuse fills, where *Ovis*/*Capra* remains the dominant taxon and dogs are only sparsely represented (Figure 5; Table 5). Pigs constitute the second most abundant taxon, followed by cattle. Although cattle rank second in terms of the NISP, pigs are more prominent when considering the MNI (Figure 5).

The skeletal representation also differs markedly between the two types of assemblages. The dogs from graves 35, 43, and 53, together with the complete sheep from grave 20, exhibit a well-balanced representation of the axial and appendicular skeleton, consistent with the deposition of complete carcasses (Table 4). In contrast, the refuse fills are characterized by a reduced representation of axial elements and an uneven distribution of anterior and posterior limb bones (Table 5). Nevertheless, when considered as a whole, the refuse assemblages include all skeletal regions in most taxa. Red deer is the only clear exception, with antlers accounting for 67.7% of the identified remains, probably reflecting their selection as raw material for the manufacture of bone tools.

Finally, it is worth noting that dogs also exhibit notable variation in body size. The individual from grave 53 could not be used to estimate shoulder height because none of its long bones was complete, although the dimensions of its preserved epiphyses are comparable to those of the dog from grave 35. The latter preserved complete tibiae with maximum lengths of 16.0 and 16.1 cm, corresponding to an estimated shoulder height of 46–47 cm according to Koudelka [67]. In contrast, the dog from grave 43 exhibits extensive diagenetic fractures that precluded osteometric analysis, although its preserved skeletal elements suggest a smaller individual. These observations indicate that at least two different body-size groups of dogs were represented among the intentional animal deposits at Humanejos.

## 5. Discussion and Conclusions

At first sight, the presence of faunal remains within funerary contexts may suggest that they formed part of symbolic practices associated with burial rituals, such as grave goods, offerings, or other forms of ritual behaviour. However, the assemblage at Humanejos demonstrates that the association between animal remains and human burials is more complex. Most of the faunal remains recovered from the graves display a taxonomic composition, mortality profiles, and taphonomic alterations comparable to those documented in domestic contexts from Copper Age settlements across the inland Iberian Peninsula. These observations indicate that a substantial proportion of the assemblage is consistent with the secondary deposition of domestic refuse within the grave fills rather than with intentional funerary deposits. Nevertheless, a smaller group of faunal remains displays characteristics consistent with deliberate deposition as part of funerary practices.

The faunal assemblage recovered from the grave fills at Humanejos closely resembles those documented in domestic contexts throughout the Middle Tagus Valley. These assemblages are generally characterized by a predominance of caprines, bovines and suids, mortality profiles dominated by adult individuals, and a relatively low representation of dogs and wild taxa [3,4,8,9,12,13,14,15]. Similar patterns have also been reported in funerary contexts such as Camino de las Yeseras [38], Aldovea [13], and other Copper Age sites [36]. Such similarities suggest that a substantial proportion of the faunal remains recovered from the graves at Humanejos may derive from domestic refuse incorporated into the pit fills. At the same time, this overlap between domestic and funerary assemblages highlights the difficulties involved in distinguishing refuse deposits from faunal remains intentionally placed as part of funerary practices.

The mortality profiles and butchery evidence further support the interpretation that much of the faunal assemblage derives from domestic refuse. The age-at-death distribution of cattle is consistent with husbandry strategies combining meat production with the exploitation of secondary products, particularly milk. In contrast, the mortality profile of pigs suggests greater emphasis on meat production, a pattern further supported by the presence of butchery marks. Caprines appear to have fulfilled a broader economic role, as their mortality profiles are compatible with the exploitation of both meat and dairy resources.

Wild taxa, particularly red deer and horse, also formed part of the subsistence economy at Humanejos. Cut marks recorded on their remains provide clear evidence of carcass processing and consumption (Table 3). In addition, shed antlers were recovered from graves 25 and 35, while the skeletal representation of red deer is dominated by fragmented antlers (Table 5). Together with the worked antler artefacts recovered from graves 34, 66, and 87, this evidence indicates that red deer also represented an important source of raw material for antler artefact production.

Dogs constitute a distinctive component of the assemblage. Most individuals reached adulthood, except for a subadult recovered from burials 53 and a young adult of burial 53. No cut marks were identified on any of the dog remains, suggesting that, unlike other taxa, they were not processed for consumption. This pattern may indicate that dogs occupied a particular position within pre-Beaker communities at Humanejos.

Their specific role remains uncertain. Nevertheless, dogs may have participated in activities such as hunting or livestock management, both of which are compatible with the economic practices inferred from the faunal assemblage. This interpretation is also consistent with the medium body size estimated for the dog from grave 35 (67). Further palaeopathological analyses may help to clarify the functions performed by these animals and provide a better understanding of their relationship with human communities during the Copper Age.

Although most of the faunal assemblage appears to derive from domestic refuse incorporated into the grave fills, a smaller group of remains reflects deliberate depositional practices associated with funerary behaviour. Such intentional animal deposits are well documented in Copper Age funerary contexts across the Iberian Peninsula, including El Tajillo del Moro (Málaga), Casa Noguera (Murcia), Monte das Cabeceiras (Beja), Camino de las Yeseras (Madrid), Gobaederra (Álava), and Cerro de la Cabeza (Ávila), among many others [3,4,8,9,12,13,14,15,89,90,91,92]. Therefore, the presence of similar deposits at Humanejos is not unexpected. Nevertheless, only nine intentional deposits were identified, occurring in 29% of the pre-Beaker burials. As described in the previous section, these deposits are characterized by the preservation of anatomical connections and the absence of anthropogenic modifications associated with carcass processing, such as butchery marks or intentional bone breakage. They are generally associated with other grave goods and were placed near the deceased. The identified deposits comprise three cattle skulls, three complete dogs, one piglet, one complete sheep, and one articulated sheep forelimb.

Nevertheless, not all faunal remains can be assigned unequivocally to either domestic refuse or intentional deposits. Several finds occupy a more ambiguous position and illustrate the interpretative difficulties inherent in funerary contexts. For example, burial 35 contained a shed antler deposited alongside the deceased, which may represent either a grave good or a piece of raw material placed within the burial. A similar situation is observed in burial 25, where a cattle horn could be interpreted either as part of the pattern of cranial deposits documented at the site or as the deposition of a raw material resource.

Another particularly complex case concerns burial 60. Two postcranial cattle remains were recovered at the same stratigraphic level as the laterally positioned individual and the associated grave goods (Figure 4). These bones were located close to the ventral region of the body and lacked cut marks, although they were fractured and disarticulated. Their interpretation therefore remains uncertain. They may represent a meat offering, a funerary deposit comparable to the piglet recovered from the same burial, or, alternatively, intrusive remains incorporated into the fill.

These examples highlight the need for greater methodological standardization in the recording and interpretation of faunal remains from funerary contexts. Future comparative and synthetic studies may help to refine the criteria used to distinguish intentional deposits, grave goods, offerings, and intrusive faunal remains, thereby improving our understanding of the diverse roles animals played in Copper Age funerary practices.

Finally, the interpretations proposed here should be regarded as preliminary and will benefit from comparison with the faunal assemblage recovered from the pre-Beaker settlement of Humanejos, which is currently under study. Such comparison between funerary and domestic contexts will provide a valuable framework for assessing similarities and differences in taxonomic composition, mortality profiles, and carcass-processing practices. In particular, it will help determine whether the patterns identified in the funerary assemblage, including the predominance of caprines in refuse deposits and the selective deposition of dogs, cattle, and pigs in funerary contexts, are specific to burial practices or reflect broader patterns of animal exploitation within the community. Ultimately, this comparison will contribute to a better understanding of the relationship between everyday economic activities and funerary behaviour in Copper Age societies of the Iberian Peninsula.

## Figures and Tables

**Table 1:** Information on the graves and their relationship with the faunal remains.

Burial	Fauna in Fill	NISP	MNI	Intentional Deposit	Human MNI	Grave Goods
13					2 adults	1 vessel
20	X	137	3	1 sheep	3 adults	1 dagger, 2 flint points
21	X	3	2		1 adult	1 vessel, 2 flint blades and 1 polished adze
25	X	79	5	1 cattle skull	6 non-adult crania	Red deer antler?
29	X	6	3		1 non-adult	1 bone awl
33	X	319	15		1 adult, 1 non-adult	
34	X	16	3		2 non-adults	
35	X	415	17	1 dog	3 adults, 1 non-adult	1 vessel, red deer antler?
43	X	120	4	1 dog	1 young adult	1 awl
44					2 adults	1 vessel
45					1 adult	2 vessels
46	X	4	1		3 adults	2 vessels, 2 flint blades, 2 flint points, 3 copper objects
47					2 adults, 2 non-adults	2 bone rods, 2 copper objects
48					2 adults	5 vessels
49					1 adult, 3 non-adults	11 vessels, 3 copper objects, 20 variscite beads
51	X	3	1		1 adult	1 vessel
52					2 adults, 1 non-adult	5 vessels and 1 copper object
53					1 adult	
SU 10982 *	X	120	1	1 dog		5 vessels
54					8 non-adults	11 vessels and 4 variscite beads
56					1 adult	
59	X	1	1		1 adult	
60	X	120	7	1 piglet, 1 cattle skull	1 adult	1 bone awl
62	X	794	18	1 cattle skull	2 adults	1 vessel, red deer antler?
63					2 adults	5 copper objects, 2 bone rods, 11 variscite beads
64					1 adult	3 vessels, 1 copper object, 60 variscite beads
65	X	34	3		2 adults	5 vessels, 4 copper objects, 63 clinochlore beads
66	X	5	1	1 sheep forelimb (humerus, radius, ulna)	2 adults	4 vessels, 7 copper objects, 1 flint point and 1 tabular flint piece
67					1 adult	1 flint point
68	X	1	1		1 adult	6 flint points
69	X	145	12		1 Adult	
70	X	103	5		1 adult cranium	
73	X	80	10		1 adult cranium	
78	X	26	4		1 Adult	
79	X	67	5		1 adult	
84					1 adult	1 flint point
86	X	39	6		1 adult cranium	
87	X	161	5		1 adult cranium	
88	X	4	1		1 adult cranium	
92	X	90	9		1 adult cranium	
93	X	209	15		1 adult cranium	
98	X	157	12		1 Adult	
99	X	437	20		Adult femur	
100	X	33	5		Adult femur	
106	X	212	10		Adult femur	

* (associated with burial 53).

**Table 2:** Taxonomic representation based on the NISP and MNI. The table also shows the percentage representation according to the NISP and MNI, as well as the percentage that is immature (non-adult individuals). The representation of each taxon by grave is provided, together with the NI (number of individuals), indicating individuals considered to form part of a possible intentional deposit in graves 20, 25, 35, 43, 53, 60, 62, and 66.

Species	Fauna in Pit Burials					Frequency			Presence in Pits		Deposits	
	NISP	MNI	Ad	Ju	In	%NISP	%MNI	% of Non-Adults MNI	No. Cases	%	NI	%
*Bos taurus*	914	30	27	0	3	23.2	14.9	11.1	22	71.0	3	33.3
*Equus ferus*	115	12	12	0	0	2.9	6.0	0	12	38.7	0	
*Ovis*/*Capra*	671	54	26	12	16		26.9	51.8	23	74.2		
*Ovis aries*	213	16	16	213	16		8.0	0	8	25.8	2	22.2
*Capra hircus*	51	6	6	51	6		3.0	0	5	16.1		
*All Ovis*/*Capra*	935	76	48	12	16	23.7	37.9	58.3	26	83.9	2	22.2
*Sus* sp.	409	34	19	8	7	10.4	16.9	78.9	18	58.1	1	11.1
*Canis familiaris*	448	16	15	1	0	11.3	8.0	6.3	15	48.4	3	33.3
*Cervus elaphus*	168	20	18	1	1	4.3	10.0	11.1	16	51.6		
*Oryctolagus* *cuniculus*	40	12	11	1	0	1.0	6.0	9.1	6	19.4		
*Capreolus* *capreoulus*	2	1	1	0	0	0.1	0.5	0	1	3.2		
Large-sized	232	0	0	0	0	5.9						
Medium-sized	21	0	0	0	0	0.5						
Small-sized	588	0	0	0	0	14.9						
Indet.	76	0	0	0	0	1.9						
Total	3948	201	152	22	27	100.0					9	

**Table 3:** Some of the bones showing cut marks in different species from the pre-Beaker animal remains of Humanejos.

Burial	Species	Anatomical Part with Cut Marks and Function	Burial	Species	Anatomical Part with Cut Marks and Function
25	*Bos*	Metacarpal -disarticulation-	33	*Ovis*/*Capra*	Scapula -defleshing-
35	*Bos*	Vertebrae -disarticulation-	35	*Ovis*/*Capra*	Mandible -disarticulation-
65	*Bos*	Metatarsal -disarticulation-	60	*Ovis*/*Capra*	Scapula -defleshing-
69	*Bos*	Radius -defleshing-	62	*Ovis*/*Capra*	Mandible -cheek meat removal-
70	*Bos*	Femur -filleting-	62	*Ovis*/*Capra*	Humerus -defleshing-
86	*Bos*	Radius -disarticulation-	62	*Ovis*/*Capra*	Femur -filleting-
87	*Bos*	Metacarpal -disarticulation-	69	*Ovis*/*Capra*	Humerus -disarticulation and defleshing-
98	*Cervus*	Humerus -defleshing-	73	*Ovis*/*Capra*	Tibia -defleshing-
98	*Sus*	Humerus -defleshing-	25	*Equus*	Metacarpal -disarticulation-
69	*Cervus*	Metatarsal -disarticulation-	92	*Cervus*	Radius -defleshing-

**Table 4:** Skeletal representation (NISP) of the intentional deposits of cattle, sheep, and dogs. * The piglet deposit is not included because only part of the skeleton was recovered during excavation.

Grave	25	60 *	62	20	66	35	43	53
NISP	*Bos taurus*	*Bos taurus*	*Bos taurus*	*Ovis aries*	*Ovis aries*	*Canis familiaris*	*Canis familiaris*	*Canis familiaris*
Horn core	26	2	40					
Cranium		3	252	11			11	2
Maxilla			2	2		2	2	2
Mandible						2	2	2
Teeth			6	10		24	16	16
Vertebrae				28		42	21	21
Ribs				32		34	12	24
Scapula				1		3	2	
Humerus				2	1	2	2	2
Radius				2	1	2	2	2
Ulna					1	2	1	2
Carpals							5	2
Metacarpal						3	6	10
Pelvis				2		1	2	2
Femur				1		2	2	2
Tibia				2		2	2	2
Fibula								2
Patella								2
Talus				2		2	2	2
Calcaneus				2		2	1	2
Tarsals				1				5
Metatarsal				2		4	5	10
Phalanges				10				6
Total	26	5	300	110	3	129	97	120

**Table 5:** Skeletal representation (NISP) and %NISP of the faunal remains recovered from the fills of the Humanejos burials, excluding the intentional animal deposits presented in Table 4.

	*Bos taurus*		*Equus ferus*		*Ovis*/*Capra*		*Sus* sp.		*Canis familiaris*		*Cervus elphus*	
	NISP	%	NISP	%	NISP	%	NISP	%	NISP	%	NISP	%
Horn core	53	9.1			3	0.4					105	67.7
Cranium	147	25.2	8	7.0	37	4.5	23	5.6	7	7.1	1	0.6
Maxilla	9	1.5	1	0.9	23	2.8	12	2.9	6	6.1	1	0.6
Mandible	22	3.8	1	0.9	55	6.7	12	2.9	10	10.2	5	3.2
Teeth	25	4.3	11	9.6	307	37.3	95	23.2	30	30.6		
Vertebrae	35	6.0	23	20.0	71	8.6	65	15.9	26	26.5	1	0.6
Ribs	45	7.7	15	13.0	54	6.6	60	14.7	6	6.1	1	0.6
Scapula	23	3.9	5	4.3	19	2.3	11	2.7	1	1.0	2	1.3
Humerus	17	2.9	6	5.2	27	3.3	19	4.6	4	4.1	6	3.9
Radius	18	3.1	6	5.2	26	3.2	10	2.4			5	3.2
Ulna	9	1.5	3	2.6	9	1.1	9	2.2	1	1.0		
Carpals	9	1.5	6	5.2	6	0.7	7	1.7			3	1.9
Metacarpal	18	3.1	7	6.1	9	1.1	8	2.0			5	3.2
Pelvis	32	5.5	1	0.9	25	3.0	5	1.2			2	1.3
Femur	27	4.6	5	4.3	31	3.8	5	1.2	1	1.0	3	1.9
Tibia	19	3.3	3	2.6	41	5.0	11	2.7	5	5.1	5	3.2
Patella											1	0.6
Talus	5	0.9	1	0.9	5	0.6	2	0.5	1	1.0	1	0.6
Calcaneus	6	1.0	2	1.7	8	1.0	2	0.5	1	1.0		
Tarsals	8	1.4	1	0.9	7	0.9	7	1.7				
Metatarsal	16	2.7	2	1.7	22	2.7	5	1.2			4	2.6
Metapodial	6	1.0	1	0.9	14	1.7	22	5.4	2	2.0		
Phalanges	34	5.8	7	6.1	23	2.8	17	4.2	1	1.0	4	2.6
Total	583	100.0	115	100.0	822	100.0	409	100.0	102	104.1	155	100.0

## Data Availability

All data are included in this manuscript, and the samples are housed at the Archaeological and Paleontological Regional Museum of Madrid.
